# Biomarkers to delineate bacteremia from candidemia remain a challenging issue

**DOI:** 10.1186/s13054-019-2720-2

**Published:** 2020-01-22

**Authors:** Patrick M. Honore, Christina David, Rachid Attou, Sebastien Redant, Andrea Gallerani, David De Bels

**Affiliations:** ICU Department, Centre Hospitalier Universitaire Brugmann, Brussels, Belgium

In their recent systematic review, Cortegiani et al. found that serum procalcitonin (PCT) concentrations were higher in patients with bacteremia as compared to candidemia [1]. Quality of data was poor and did not allow to use PCT alone to differentiate bacteremia from candidemia [1]. While we agree with their findings regarding patients with positive hemocultures in the ward, we would like to address the results of the patients with positive hemocultures in the intensive care unit (ICU) [1]. In a randomized controlled study (RCT) comparing the clinical manifestations of septic shock caused by bacteria or *Candida* spp., the rate of acute kidney injury (AKI) defined by a creatinine above 3.5 mg/dl or the need of any form of renal replacement therapy (RRT) was around 20% in the bacteria group versus above 40% in the candida group, while having similar Acute Physiology and Chronic Health Evaluation (APACHE) II scores [2]. Accordingly, the rate of RRT in the candidemia group will be twice as high as compared to the bacteremia group [2]. It is plausible that there are similar trends in the Cortegiani study [2]. PCT has an approximate molecular weight of 14.5 kDa [3]. The contemporary continuous RRT (CRRT) membranes are able to remove molecules as large as 35 kDa [3]. Hence, most of the PCT mass will be eliminated by convective flow [3], but adsorption also contributes to elimination if using new highly adsorptive membranes (HAM) [4]. Accordingly, imbalance between the use of CRRT in the two cohorts (bacteremia versus candidemia) will have an important impact upon the values of PCT in each cohort. PCT levels may therefore be affected not only by the type of pathogen but also by the incidence of RRT. A future study with a focus on the performance of the currently known sepsis biomarkers among those who receive CRRT is urgently needed [4]. As alluded too by Cortegiani et al., beta-d-glucan (BDG) could be a very good candidate associated to PCT. Indeed, BDG could be even a better candidate as its molecular weight ranges from several hundred thousand to 10 million daltons and does not pass through any membrane [4, 5]. Finally, we would like to add that BDG can be also be falsely elevated in case of gastrointestinal colonization of *Candida albicans* that increases serum BDG without candidemia [5].

## Authors’ response: Procalcitonin, candidemia, and CRRT: more research is needed but do not forget pathophysiology

Andrea Cortegiani, Mariachiara Ippolito and Antonino Giarratano

We would like to thank Dr. Honore et al. for discussing the potential role of acute kidney injury (AKI) and continuous renal replacement therapy (CRRT) as potential confounders on the different values of procalcitonin (PCT) in patients with bacteremia versus candidemia in the intensive care unit (ICU) in our systematic review [1]. We evaluated the included studies conducted in ICU setting, and we can confirm that no study specifically reported the rate of CRRT in both bacteremia and candidemia cohorts [1]. One study generally reported the proportion of patients receiving “hemodialysis” at the moment of infection, without significant difference (10% candidemia group vs 14% bacteremia group) [6]. Indeed, PCT values in patients with bloodstream infection from bacteria and *Candida* spp. receiving CRRT and blood purification techniques should be addressed in future studies. Another interesting aspect would be the impact of CRRT in the trend of PCT values in these patients, since almost all the included studies reported the values at the beginning of the diagnostic process [1]. However, the pathophysiology of the immune response and inflammatory response seems to be different in patients with candidemia, with signs of immune cell exhaustion, suppressive immunophenotype of T cells, and concomitant downregulation of positive co-stimulatory molecules [7–9]. These findings may be considered the main potential mechanisms for the different PCT level at the moment of the diagnostic process even in the ICU patients.

## Data Availability

Not applicable.

## Abbreviations

PCT: Procalcitonin
ICU: Intensive care unit
AKI: Acute kidney injury
SA-AKI: Sepsis-associated AKI
APACHE II: Acute Physiology and Chronic Health Evaluation II
RRT: Renal replacement therapy
CRRT: Continuous renal replacement therapy
HAM: Highly adsorptive membranes
BDG: Beta-d-glucan
